# Trimodal therapy with high-dose-rate brachytherapy and hypofractionated external beam radiation combined with long-term androgen deprivation for unfavorable-risk prostate cancer

**DOI:** 10.1007/s00066-021-01784-3

**Published:** 2021-04-28

**Authors:** Keiichiro Mori, Hiroshi Sasaki, Yuki Tsutsumi, Shun Sato, Yuki Takiguchi, Shun Saito, Eriko Nishi, Gen Ishii, Toshihiro Yamamoto, Yusuke Koike, Jun Miki, Tatsuya Shimomura, Takahiro Kimura, Kenta Miki, Shahrokh F. Shariat, Hiroyuki Takahashi, Manabu Aoki, Shin Egawa

**Affiliations:** 1 Department of Urology, The Jikei University School of Medicine, 3-25-8, Nishi-Shimbashi, Minato-ku, 105-8461 Tokyo, Japan; 2 Department of Urology, Medical University of Vienna, Vienna, Austria; 3 Department of Radiology, The Jikei University School of Medicine, Tokyo, Japan; 4 Department of Pathology, The Jikei University School of Medicine, Tokyo, Japan; 5 Karl Landsteiner Institute of Urology and Andrology, Vienna, Austria; 6 Division of Urology, Department of Special Surgery, The University of Jordan, Amman, Jordan; 7 Department of Urology, Weill Cornell Medical College, New York, NY USA; 8 Department of Urology, University of Texas Southwestern, Dallas, TX USA; 9 Department of Urology, Second Faculty of Medicine, Charles University, Prague, Czech Republic; 10 Institute for Urology and Reproductive Health, I.M. Sechenov First Moscow State Medical University, Moscow, Russian Federation; 11 European Association of Urology Research Foundation, Arnhem, The Netherlands

**Keywords:** National Comprehensive Cancer Network, Very high risk, High risk, Retrospective, Prognostic model

## Abstract

**Purpose:**

To assess the outcomes of high-dose-rate (HDR) brachytherapy and hypofractionated external beam radiation therapy (EBRT) combined with long-term androgen deprivation therapy (ADT) in very-high-risk (VHR) versus high-risk (HR) prostate cancer (PCa), as defined in the National Comprehensive Cancer Network (NCCN) criteria.

**Methods:**

Data from 338 consecutive HR or VHR PCa patients who had undergone this tri-modal therapy between 2005 and 2018 were retrospectively analyzed. Biochemical recurrence (BCR)-free, progression-free, overall, and cancer-specific survival (BCRFS/PFS/OS/CSS) rates were analyzed using the Kaplan–Meier method and Wilcoxon test. Cox regression models were used to evaluate candidate prognostic factors for survival. C‑indexes were used to assess model discrimination.

**Results:**

Within a median follow-up of 84 months, 68 patients experienced BCR, 58 had disease progression including only 3 with local progression, 27 died of any cause, and 2 died from PCa. The 5‑year BCRFS, PFS, OS, and CSS rates were 82.2% (HR 86.5%; VHR 70.0%), 90.0% (HR 94.3%; VHR 77.6%), 95.7% (HR, 97.1%; VHR, 91.8%), and 99.6% (HR, 100%; VHR, 98.0%), respectively. In multivariable analyses that adjusted for standard clinicopathologic features, the risk subclassification was associated both PFS and OS (*p* = 0.0003 and 0.001, respectively). Adding the risk subclassification improved the accuracy of models in predicting BCRFS, PFS, and OS.

**Conclusion:**

While the outcome of this trimodal approach appears favorable, VHR PCa patients had significantly worse oncological outcomes than those with HR PCa. The NCCN risk subclassification should be integrated into prognostic tools to guide risk stratification, treatment, and follow-up for unfavorable PCa patients receiving this trimodal therapy.

**Supplementary Information:**

The online version of this article (10.1007/s00066-021-01784-3) contains supplementary material, which is available to authorized users.

## Introduction

In the 2014 version, the National Comprehensive Cancer Network (NCCN) guidelines for clinically localized prostate cancer (PCa) refined their definition of unfavorable-risk disease into very-high-risk (VHR) and high-risk (HR) subsets [1]. The criteria for VHR include clinical stage ≥T3b or primary Gleason pattern 5 or ≥5 biopsy cores with a Gleason score (GS) of 8–10. The clinical management of these patients is complex, given that there are multiple options to deliver the optimal local control with the lowest risk of metastatic relapse [2].

Several treatment options are currently available for definitive radiotherapy in unfavorable-risk PCa [1]. In addition to conventional external beam radiation therapy (EBRT) such as 3D conformal radiotherapy and intensity-modulated radiotherapy (IMRT), brachytherapy boost with low-dose-rate or high-dose-rate (HDR-BT) has become a decent option. Dose escalation offers a greater benefit in terms of biochemical recurrence-free survival (BCRFS), especially in higher-risk PCa [3].

HDR-BT is a widely used strategy for unfavorable-risk or locally advanced PCa that allows dose escalation while minimizing toxicity [4–6]. The combination of HDR-BT and EBRT is expected to deliver higher radiation doses more safely and precisely than EBRT alone, thus representing a viable approach [4, 7, 8]. It has also been shown that radiation monotherapy in unfavorable-risk PCa offers only limited efficacy [3]. Multiple randomized trials with conventional EBRT dosage have demonstrated not only an improved rate of biochemical control, but also superior overall survival (OS) when supported with the use of long-term androgen deprivation therapy (ADT) as compared to treatment with each modality alone [9–12]. What is still unknown, however, is whether ADT is necessary in all HR and/or VHR patients treated with dose-escalated irradiation.

In this study, patients with unfavorable-risk PCa were treated with HDR-BT combined with hypofractionated EBRT and long-term ADT. Given that there is a paucity of evidence about the benefit of this trimodal approach, especially in the VHR PCa subpopulation, the purpose of this study was to report treatment outcomes in patients with VHR PCa compared to those with HR. In addition, we wanted to identify prognostic factors that would help us counsel patients with regards to the outcomes of the trimodal therapy in VHR and HR PCa patients.

## Materials and methods

### Patients

This retrospective study included 430 consecutive patients with histologically proven adenocarcinoma of the prostate and no evidence of distant metastasis, who were treated with HDR-BT at The Jikei University hospital between May 2005 and February 2018. Patients with missing data and/or inadequate follow-up (≤30 months) and/or at intermediate risk (*n* = 92) were excluded from the analysis. As per the study protocol requiring 2 years of adjuvant ADT, those followed up for a short period were excluded as being still under the influence of ADT and therefore likely confounding the oncological outcome evaluation. All the remaining 338 were stratified into two groups based on the NCCN risk grouping (HR, *n* = 245; VHR, *n* = 93). High-risk tumors were those with ≥T3a, a GS of 8–10, or a prostate‐specific antigen (PSA) >20 ng/ml. Those with VHR category included clinical stages with ≥T3b, a primary Gleason pattern of 5, or ≥5 biopsy cores with a GS of 8–10 [1]. Magnetic resonance imaging was used to determine T stages. Treatment outcomes were compared between the two groups. This study was conducted with the approval of the Institutional Review Board of The Jikei University School of Medicine, Tokyo, Japan (32-121 (10197))

### Treatment protocol and follow-up

Our HDR-BT protocol required all patients to receive 6 months of neoadjuvant ADT followed by HDR-BT as interstitial irradiation. This was followed by additional EBRT, with adjuvant ADT being continued for 2 years after HDR-BT. The study protocol also stipulated that EBRT be initiated 1 to 2 weeks following HDR-BT. ADT included a Gonadotropin releasing hormone (GnRH) agonist alone, either with 6 months depot of leuprorelin acetate 22.5 mg or 3 months formulation of goserelin acetate 10.8 mg, and, in some cases, combined with bicalutamide 80 mg/day. GnRH agonist monotherapy was generally applied for adjuvant use. Details of our irradiation protocol have been reported previously [13]. Briefly, for prostatic HDR-BT, the planning target volume (PTV), i.e., the gross target volume with a 4-mm margin around the prostate (except a 2-mm margin only on the rectum), was irradiated at the prescribed dose. The total radiation dose delivered with HDR-BT increased with advances over the years in radiation technology. Until July 2007, it involved treating the PTV with HDR-BT twice daily at a dose of 5 gray (Gy) and then at a dose of 6 Gy 6 hours later, followed by EBRT at a dose of 45 Gy (3 Gy/fractions [fx]) to the prostate (protocol 1; biological effective dose [BED] 153.75 Gy, equivalent dose in 2‑Gy fractions [EQD2] 76.88 Gy). Since August 2007, HDR-BT was administered at a once-daily dose of 9 Gy for 2 days (9 Gy × 2 fx, totaling 18 Gy), followed by EBRT at a dose of 40 Gy (2.5 Gy/fx) to the prostate (protocol 2; BED 189 Gy, EQD2 94.5 Gy). From July 2013 onwards, EBRT was delivered at a dose of 40 Gy (2.0 Gy/fx) extended to the prostate, seminal vesicles, and pelvic lymph nodes (protocol 3; BED 199 Gy, EQD2 99.5 Gy). From June 2016 onwards, EBRT was replaced by IMRT (protocol 4; BED 199 Gy, EQD2 99.5 Gy). An α/β ratio of 2.0 Gy was used for BED and EQD2 calculations. In addition, the medians (ranges) of D90 and V100 for PTV and prostate, D2cc for rectum, and dose constraints for urethra are summarized in Supplementary Table 1.

All patients were followed according to the institutional protocol and local guidelines at the time. Generally, follow-up visits included physical examination, serum chemistry evaluation including PSA, and an assessment of genitourinary or gastrointestinal adverse events. Time to OS was defined as the time from HDR-BT initiation to the occurrence of all-cause mortality, as confirmed by the hospital records and/or death certificates. Time to CSS was defined as the time from HDR-BT initiation to the occurrence of PCa mortality. Disease progression was defined as local recurrence, lymph node/distant metastasis as confirmed on CT, MRI, or bone scans, and/or death. The definition of BCR following trimodal therapy was consistent with the definition of Phoenix/American Society for Radiation Oncology [14]. All patients were assessed according to the Prostate Cancer Working Group 2 criteria. Records of adverse events (AEs) as defined by the Common Terminology Criteria for Adverse Events criteria version 4 were also reviewed.

### Statistical analysis

The treatment groups were compared for patient demographics using the *t*-test and chi-squared (χ^2^) test. CSS, OS, PFS, and BCRFS were graphically visualized using the Kaplan–Meier method. Difference between groups was assessed using the Wilcoxon test. Cox regression model was performed to compare the primary outcomes between the treatment groups. The predictors of these outcomes were tested using a multivariable Cox model based on the following variables: radiation protocol, patient age, baseline PSA levels, biopsy GS, clinical T stage, NCCN risk subclassification, PSA levels at HDR-BT initiation, PSA nadir, and time to PSA nadir. Age, baseline PSA, PSA at HDR-BT initiation, PSA nadir, and time to PSA nadir were treated as continuous values, the remaining parameters as categorical. Discrimination of models was evaluated using Harrel’s concordance index. Optimal cutoff values were determined using receiver operating curve analysis. Lymph node RT was performed only in protocols 3 and 4. These protocols are more aggressive treatment in terms of lymph node RT compared to protocol 1 and protocol 2. Therefore, we compared HR and VHR patients treated with the same BED and EQD 2 (protocol 1, protocol 2, and protocol 3 plus protocol 4) as subanalyses using the Wilcoxon test in terms of OS, PFS, and BCRFS. Statistical analyses were performed using GraphPad Prism (version 5, GraphPad Software, USA) and Stata (Version 14, StataCorp, USA). A *p-*value of less than 0.05 was considered to indicate significance and all tests are two sided.

## Results

### Patient demographics

A total of 338 patients (HR 245; VHR 93) were included in this retrospective analysis. Table 1 summarizes the clinicopathologic characteristics of the patients. Median (range) follow-up was 84 (30–180), 96 (30–180), and 60 (30–171) months in the overall (*n* = 338), HR (*n* = 245), and VHR (*n* = 93) patients, respectively. Patients in the VHR subgroup had higher PSA values, more advanced clinical T stages, and higher GS at baseline compared to those in the HR subgroup (*P* < 0.05). While VHR patients appeared to be treated more recently with protocol 3 or 4 (from July 2013 onwards), many in the HR group underwent protocol 1 or 2 until June 2013.

**Table 1:** Patient demographics

	All	High risk	Very high risk	*P*-value
*N*	338	245	93	–
*Age (years)*	69 (35–82)	69 (48–82)	69 (35–82)	0.69
*Initial PSA (ng/ml)*	25.09 (1.2–702.86)	25.27 (1.2–365.89)	24.91 (4.5–702.86)	0.023
*Clinical T stage 3≧ (n)*	116 (34.3%)	62 (25.3%)	54 (58.1%)	<0.001
*Gleason score 8≧ (n)*	274 (81.1%)	185 (75.5%)	89 (95.7%)	<0.001
*PSA at HDR-BT (ng/ml)*	0.08 (0.001–277.55)	0.09 (0.001–277.55)	0.07 (0.001–211.63)	0.66
*PSA nadir (ng/ml)*	0.001 (0.001–2.53)	0.001 (0.001–2.53)	0.001 (0.001–2.53)	0.14
*Time to PSA nadir (months)*	4 (0–85)	5 (0–85)	4 (0–75)	0.83
*Protocol (number)*				0.001
1	64 (18.9%)	56 (22.9%)	8 (8.6%)	
2	177 (52.4%)	132 (53.9%)	45 (48.4%)	
3	62 (18.3%)	37 (15.1%)	25 (26.9%)	
4	35 (10.4%)	20 (8.2%)	15 (16.1%)	
*Follow up (months)*	84 (30–180)	96 (30–180)	60 (30–171)	<0.001

Values are expressed in median (range)

*HDR-BT* high-dose-rate brachytherapy, *PSA* prostate-specific antigen

### Survival outcomes

**Fig. 1:**
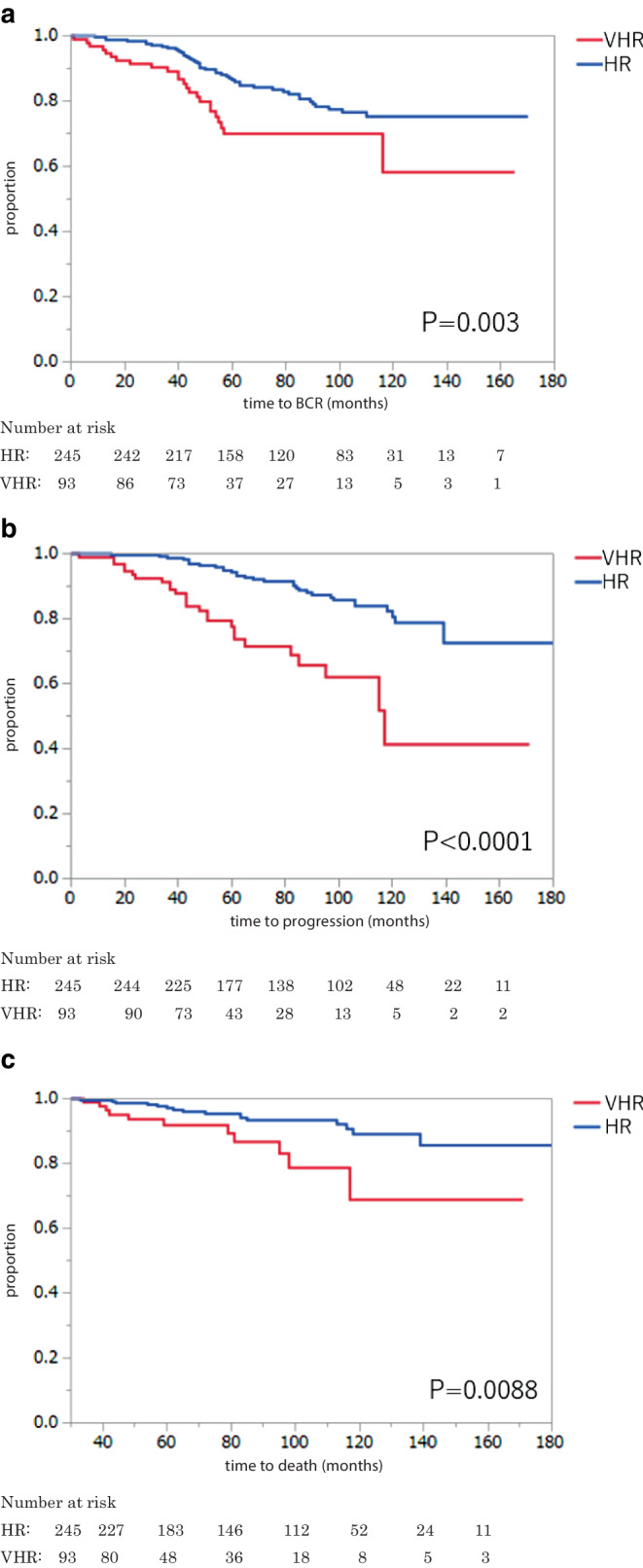
Survival analysis based on NCCN risk subclassification (very high risk [*VHR*] vs. high risk [*HR*]). **a** Biochemical recurrence (*BCR*)-free survival. **b** Progression-free survival. **c** Overall survival

Within a median follow-up of 84 months, 68 patients (20.1%) experienced BCR, 58 (17.2%) developed disease progression (local in 3 patients with prostatic in 2 HR and bladder neck invasion in 1 VHR), 27 (8.0%) died of any cause, and 2 (0.6%) died from PCa. BCRFS, PFS, OS, and CSS rates in the overall cohort were 82.2%, 90.0%, 95.7%, and 99.6%, respectively, at a 5-year follow-up; 75.1%, 81.4%, 91.1%, and 99.6% at 8‑year, and 71.6%, 73.4%, 85.5%, and 98.4% at a 10-year follow-up, respectively. BCRFS, PFS, OS, and CSS rates in the HR cohort were 86.5%, 94.3%, 97.1%, and 100% at the 5‑year follow-up. These figures in the VHR cohort were much lower, with 70.0%, 77.6%, 91.8%, and 98.0% at the 5‑year follow-up. The NCCN subclassification was significantly associated with BCRFS, PFS, and OS in the Kaplan–Meier and Wilcoxon test (*p* = 0.003, *p* < 0.0001, and *p* = 0.0088, respectively; Fig. 1). The radiation protocol was, however, not significantly associated with survival outcomes (data not shown). Table 2 shows the results of univariable and multivariable Cox proportional hazard regression analyses in the overall cohort. In the univariable analysis, the NCCN risk subclassification was significantly associated with BCRFS, PFS, and OS (*p* = 0.015,* p* < 0.0001, and* p* = 0.011, respectively). In the multivariable analysis, after adjusting for the effects of standard clinicopathologic features, the NCCN risk subclassification remained an independent predictor for both PFS and OS (*p* = 0.0003 and* p* = 0.001, respectively). The integration of this parameter improved the accuracy of the models in predicting BCRFS (accuracy 66%) by 2 points, PFS (accuracy 69%) by 4 points, and OS (accuracy 71%) by 8 points compared with models including all significant parameters except the NCCN risk subclassification. The median (range) PSA nadir was 0.001 (0.001–2.53), 0.001 (0.001–2.53), and 0.001 (0.001–2.53) ng/ml in the overall, HR, and VHR patients, respectively. Posttreatment PSA nadir was significantly associated with BCRFS and PFS in the multivariable analyses. A PSA nadir ≤0.01 ng/mL displayed better BCRFS, PFS, and OS than a PSA nadir ≥0.02 ng/mL in the Wilcoxon analysis (*p* < 0.0001, *p* < 0.0001, and *p* = 0.031, respectively; Supplementary Fig. 1). A total of 267/326 (81.1%) patients had a PSA nadir ≦0.01 ng/ml. Sensitivity, specificity, positive predictive value (PPV), and negative predictive value (NPV) were 14.2%, 55.9%, 59.4%, and 12.6% for BCR, respectively. For disease progression, sensitivity, specificity, PPV, and NPV were 13.1%, 66.1%, 63.6%, and 14.4%, respectively. For overall mortality, sensitivity, specificity, PPV, and NPV were 6.4%, 88.1%, 70.8%, and 17.2%, respectively. The median (range) time to PSA nadir was 4 (0–85), 5 (0–85), and 4 (0–75) months in the overall, HR, and VHR patients, respectively. Time to PSA nadir was also independently associated with BCRFS. Patients with a time to PSA nadir ≤6 months displayed better BCRFS than those with a time to PSA nadir ≥7 months in the in the Wilcoxon analysis (*p* = 0.0055, Supplementary Fig. 2). A total of 179/311 (57.6%) had a time to PSA nadir ≦6 months. Sensitivity, specificity, PPV, and NPV were 13.4%, 72.7%, 40.0%, and 38.2% for BCR, respectively.

**Table 2:** Cox regression analysis for survival outcomes

	Biochemical recurrence-free survival				Progression-free survival				Overall survival			
	Univariable		Multivariable		Univariable		Multivariable		Univariable		Multivariable	
	HR (95% CI)	*P-*value	HR (95% CI)	*P-*value	HR (95% CI)	*P-*value	HR (95% CI)	*P-*value	HR (95% CI)	*P-*value	HR (95% CI)	*P-*value
Protocol	0.82 (0.57–1.16)	0.69	–	–	0.83 (0.55–1.25)	0.77	–	–	0.40 (0.20–0.81)	0.030*	–	0.21
Age	0.96 (0.93–1.00)	0.027*	0.97 (0.94–1.00)	0.064	0.99 (0.96–1.03)	0.70	–	–	1.04 (0.98–1.11)	0.21	–	–
Initial PSA	1.00 (1.00–1.01)	0.12	–	–	1.00 (1.00–1.00)	0.97	–	–	1.00 (0.99–1.00)	0.84	–	–
Clinical T stage Reference; 2≦	1.00 (0.57–1.68)	0.99	–	–	0.98 (0.52–1.77)	0.95	–	–	1.06 (0.41–2.48)	0.89	–	–
Gleason score Reference; 8≦	2.58 (1.57–4.38)	0.0002*	2.36 (1.31–4.37)	0.0042*	2.13 (1.26–3.68)	0.0046*	1.52 (0.84–2.82)	0.17	1.13 (0.53–2.43)	0.75	–	–
NCCN risk Reference; high	1.91 (1.14–3.12)	0.015*	1.29 (0.71–2.29)	0.39	3.66 (2.14–6.22)	<0.0001*	3.01 (1.66–5.44)	0.0003*	2.91 (1.30–6.30)	0.011*	3.56 (1.63–7.75)	0.001*
PSA at HDR-BT	1.01 (1.01–1.02)	0.0018*	1.01 (1.00–1.02)	0.073	1.00 (0.99–1.01)	0.45	–	–	0.92 (0.45–1.01)	0.40	–	–
Time to PSA nadir	1.02 (1.01–1.04)	0.0055*	1.02 (1.00–1.04)	0.042*	1.00 (0.98–1.02)	0.73	–	–	1.00 (0.97–1.02)	0.98	–	–
PSA nadir	3.71 (1.65–6.40)	0.0049*	2.83 (1.40–5.70)	0.029*	4.41 (2.23–8.72)	0.0032*	3.98 (1.68–7.39)	0.005*	2.29 (0.01–9.36)	0.60	–	–
C index with NCCN risk	0.66				0.69				0.71			
C index without NCCN risk	0.64				0.65				0.63			

*CI* confidential interval, *HDR-BT* high-dose-rate brachytherapy, *HR* hazard ratio, *NCCN* National Comprehensive Cancer Network, *PSA* prostate-specific antigen

*statistically significant *p*-value

### Survival outcomes based on BED and EQD2

While we could not compare OS between HR and VHR patients as those treated according to protocols 3 plus 4 had been followed up for so short a period that no death events had occurred among these patients, there was a significant difference in PFS and BCRFS between HR and VHR patients in this group (Supplementary Fig. 3). There was also a significant difference in PFS and OS between HR and VHR patients treated according to either protocol 1 or 2 (Supplementary Fig. 3). Patient characteristics by protocol are summarized in Supplementary Table 2.

### Adverse events

No grade 4 or higher AEs were observed. All grade 3 hematuria caused by radiation cystitis occurred in patients with HR disease (*n* = 6). All but one patient were successfully treated with transurethral elective cauterization. The remaining patient subsequently received hyperbaric oxygen therapy. No clear relevance of treatment protocol was observed. Two patients were treated with protocol 1, three with protocol 2, and one with protocol 3. None of the protocol 4 patients experienced grade 3 AEs. The overall incidence of AEs increased with increasing BED but decreased with the initiation of protocol 4 (Table 3).

**Table 3:** Adverse events (AEs)

	Grade 1 or 2	Grade 3	Total
*AEs based on the National Comprehensive Cancer Network risk classification*			
High risk	77/245 (31.4%)	6/245 (2.4%)	83/245 (33.9%)
Very high risk	27/93 (29.0%)	0/93 (0%)	27/93 (29.0%)
*AEs based on protocol*			
Protocol 1	5/64 (7.8%)	2/64 (3.1%)	7/64 (10.9%)
Protocol 2	65/177 (36.7%)	3/177 (1.7%)	68/177 (38.4%)
Protocol 3	26/62 (41.9%)	1/62 (1.6%)	27/62 (43.5%)
Protocol 4	8/35 (22.9%)	0/35 (0%)	8/35 (22.9%)

## Discussion

This study investigated the outcomes of trimodal therapy with HDR-BT and hypofractionated EBRT combined with long-term ADT for VHR PCa compared to HR PCa. Study results indicate that patients with VHR PCa suffered from worse BCRFS, PFS, and OS than those with HR PCa. These findings are of interest given the paucity of studies assessing the outcomes of this approach for VHR PCa. Interestingly, even in VHR patients, a 5-year BCRFS of 70.0% could be achieved with this combination therapy. Although BCR is likely to continue to occur with further follow-up, our results appear promising compared with other HDR-BT studies in unfavorable-risk PCa, where the BCRFS rates varied between 59 and 94% with or without hormonal therapy (with more than 5‑year median follow-up) [2, 15, 16]. Indeed, reported outcomes of HDR-BT with and without ADT have been highly variable, needing external validation. Regarding protocol, while the treatment protocol had no impact in Cox regression analyses, VHR patients appeared to be treated more recently with protocol 3 or 4 (i.e., involving more aggressive treatment in terms of lymph node RT). Thus, we compared HR and VHR patients treated with the same BED and EQD2 in sub-analyses and demonstrated a similar trend to those between HR and VHR patients in the overall study population, suggesting the protocol-independent usefulness/validity of the NCCN risk classification.

Another point worth making is that given that the majority of patients suffered distant failure (with only three having experienced local failure) in this study, it remains still unclear whether escalation of the irradiation dose to the prostate may favorably affect distant failure. One possible hypothesis could be that a higher proportion of activated T cells versus a lower proportion of regulatory T cells in men after brachytherapy may contribute to remission being maintained as well as to delays with distant failure through immunomodulatory mechanisms [17]. However, while trimodal therapy was shown to produce outcomes in VHR patients as favorable in other studies as in ours, these patients tended to have metastases at disease progression [16, 18]. Thus, it remains to be further examined whether or not escalation of the irradiation dose to the prostate improves distant failure.

Combining ADT with conventional dose EBRT appears necessary, given that this has been conclusively shown to improve OS in unfavorable-risk diseases [9]. However, the role of ADT and, more specifically, optimal duration of ADT including neoadjuvant and adjuvant therapy in conjunction with dose-escalated irradiation therapy remains unclear. In contrast to our study, which stipulated 6 months of neoadjuvant ADT and 24 months of adjuvant ADT, earlier studies implemented adjuvant ADT over a varying range of durations with none being as long as 36 months, but none implemented neoadjuvant ADT for as long as 6 months. Our study found more favorable outcomes in patients with HR disease compared to those with VHR disease, suggesting that shortening the duration of ADT in HR, without compromising outcomes, should be considered in well-designed trials. A previous trimodal therapy study that used ≥6 months of neoadjuvant and 36 months of adjuvant ADT achieved more favorable oncologic outcomes than our studied patients with VHR (*n* = 82; 5‑year BCRFS 81.9%; PFS 92.1%; and OS 93.3%) [16]. At least a year of extension in ADT use (30 in our study versus 42 months) seems to add value in VHR patients, with a 10–15% margin in BCRFS and PFS. Whether this would ultimately translate into better OS, however, remains to be seen. To date, no randomized trials of ADT combined with HDR-BT/EBRT have been completed. Further studies are necessary to explore whether ADT improves survival and, if it does, to define the optimal duration when combined with HDR-BT and EBRT. Moreover, further research is warranted to determine the optimal durations of not only total ADT but also of neoadjuvant and adjuvant ADT. Trade-offs in terms of toxicity using such an approach must also be taken into consideration.

A high PSA nadir was found to be significantly associated with poor PFS and BCRFS. A PSA nadir of ≤0.01 ng/mL was a significant independent predictor of a better outcome. Previous studies have suggested that the PSA nadir after prostate radiotherapy is well correlated with prognosis, including CSS [19–23]. The threshold of PSA nadir varies depending on the extent of local involvement, the type of radiotherapy administered, and the duration of ADT. The PSA nadir suggested in this study to predict clinical outcomes was extremely low compared with those reported in previous studies, which ranged from 0.02 to 1.5 ng/mL [19–23]. Combined HDR-BT and EBRT allow radiation doses to be delivered more precisely and intensely, and thus more effectively than conventional EBRT, and are indicated even in patients with extracapsular extensions and seminal vesicle invasion. Precise local control induced by this dose intensification might account in part for the lower PSA nadir threshold seen in this study. Time to PSA nadir appears to be another crucial predictor for BCRFS. Patients who do not fulfill these conditions might require careful observation and additional treatment, given that they might be at greater risk of treatment failure. In summary, “early (within 6 months after treatment)” and “complete (PSA ≤0.01 mg/ml)” PSA decline portend better outcomes and provide important information for clinical decision-making regarding therapeutic considerations and patient counseling.

The incidence of grade 3 toxicities (1.8%) in this study was relatively low compared to previous reports (0–14%) with all patients but one being managed successfully with transurethral electrocauterization [2, 15]. A decreasing trend in grade 3 and any-grade toxicities was observed after the introduction of IMRT at our institution (2.3 versus 0% and 33.7 versus 22.9%, respectively); longer follow-up is, however, needed to draw any meaningful conclusions.

Conventional imaging modalities have a limited sensitivity to detect microscopic extrapelvic PCa [24, 25]. This is particularly relevant as such lesions likely exist outside the conventional clinical target volume [25]. Hence, patients in the present study may have harbored prostate specific membrane antigen (PSMA)-positive lesions lying outside of the irradiated areas. This highlights the consistent necessity for more precise staging as well as for individualized, tailored management in this era of PSMA positron emission tomography. Furthermore, this study also showed that only three patients had local disease progression, thus highlighting the importance of controlling micrometastases to improve treatment outcomes in patients with unfavorable PCa. The future focus of study in this unfavorable PCa population must include more accurate staging combined with a smart use of novel systemic agents such as androgen receptor axis targeting drugs, chemotherapy, ^177^Lu-PSMA theranostics, and stereotactic body radiotherapy in a sandwich therapeutic strategy.

While this study offers several potentially relevant findings, it is not devoid of limitations. First, the technical modifications of radiotherapy, inconsistent durations of neoadjuvant ADT, and liberal use of nonsteroidal anti-androgen agents in the study population together with the potentially variable sustained castrate-level testosterone levels after discontinuation of ADT might have influenced the interpretation of the data. Second, patient follow-up and subsequent treatment were not standardized because of the retrospective nature of the study. Third, the median follow-up duration of 86 months and the low number of deaths undermine any conclusions regarding mortality. The median follow-up for patients with VHR disease was much shorter than in those with HR diseases. In addition, our single-center experience might not be generalizable to other institutions. Survival outcome data from multiple centers involving larger patient populations is needed to critically determine the utility of NCCN risk subclassification. Fourth, despite being a major prognostic factor, PSA bounce was not included in our analysis, because our study protocol meant that adjuvant ADT may still have been effective enough to affect the occurrence of PSA bounces when the patients were most likely to have been susceptible to them [26, 27]. Fifth, the AEs reported in this study were not presented as early/late toxicities due to a paucity of relevant data in our database. Moreover, none of the ADT-related toxicities were systematically monitored and registered in this study. This critical issue should be prospectively elucidated in more systematic schemes to appraise the long-term risks and trade-offs. Lastly, in this paper focused on trimodal therapy, it should be of interest to compare this modality with radical prostatectomy (RP) in patients with unfavorable-risk PCa. Kishan et al. retrospectively compared treatment outcomes with RP versus EBRT plus ADT versus EBRT plus BT plus ADT (trimodal therapy), reporting that trimodal therapy using HDR is superior to other modalities in patients with GS 9–10 PCa with respect to mortality from PCa and distant metastasis [28]. Despite being a large cohort study, this study has a major limitation due to the incomplete surgical resections and/or lack of adjuvant therapy implemented in its RP cohort. Thus, this issue warrants further study.

In conclusion, our data demonstrate that HDR-BT, hypofractionated EBRT, and long-term ADT in combination provide favorable oncological outcomes with acceptable, readily manageable AE for both VHR and HR PCa patients. However, patients with VHR PCa had significantly worse oncological outcomes than those with HR disease. NCCN risk subclassification should be incorporated into prognostic tools to guide treatment planning and patient follow-up in unfavorable-risk patients treated with this trimodal approach. Finally, well-designed prospective studies with long-term follow-up are warranted to validate all these findings and improve upon them.

## Supplementary Information

Supplementary Table 1

Supplementary Figure 1

Supplementary Figure 2

Supplementary Figure 3

Supplementary Table 2
