# Supplementary material for: Trimodal therapy with high-dose-rate brachytherapy and hypofractionated external beam radiation combined with long-term androgen deprivation for unfavorable-risk prostate cancer
Source: Strahlenther Onkol. 2021 Apr 28;197(11):976–85. doi: 10.1007/s00066-021-01784-3 (PMC8547210; doi:10.1007/s00066-021-01784-3)
Supplement: Supplementary file 1 — Supplementary Table 1 [file 66_2021_1784_MOESM1_ESM.docx]

Supplementary Table 1

D90, V100 for planning target volume (PTV) and prostate, D2cc for rectum and dose constraints for urethra

|  | Protocol 1 | | Protocol 2, 3, 4 |
| --- | --- | --- | --- |
|  | 1st brachytherapy | 2nd brachytherapy |  |
| D90 for prostate  median (range) | 6.23  （6.15～6.88） | 7.59  （7.28～8.01） | 11.33  （11.09～12.24） |
| D90 for PTV  median (range) | 5.15  （4.96～5.88） | 6.26  （5.98～6.78） | 9.32  （8.87～10.75） |
| V100 for prostate  median (range) | 99.51  （96.8～100） | 99.34  （97.8～100） | 99.42  （98.5～100） |
| V100 for PTV  median (range) | 91.9  （88.3～97.63） | 92.01  （87.9～99.1） | 92.15  （89～98.58） |
| D2cc for rectum  median (range) | 3.82  （3.01～4.21） | 4.32  （3.80～5.03） | 6.38  （3.89～9.16） |
| Dose constraints for urethra D90% | >4.8Gy | >5.7Gy | >8.55Gy |
| Dose constraints for urethra Dmax | <6.25Gy | <7.5Gy | <11.25Gy |
