# Supplementary material for: Trimodal therapy with high-dose-rate brachytherapy and hypofractionated external beam radiation combined with long-term androgen deprivation for unfavorable-risk prostate cancer
Source: Strahlenther Onkol. 2021 Apr 28;197(11):976–85. doi: 10.1007/s00066-021-01784-3 (PMC8547210; doi:10.1007/s00066-021-01784-3)

Supplementary Figure 1

Survival analysis based on PSA nadir (PSA nadir ≦0.01ng/ml vs ≧0.02ng/ml)

A) Biochemical recurrence free survival (BCRFS)

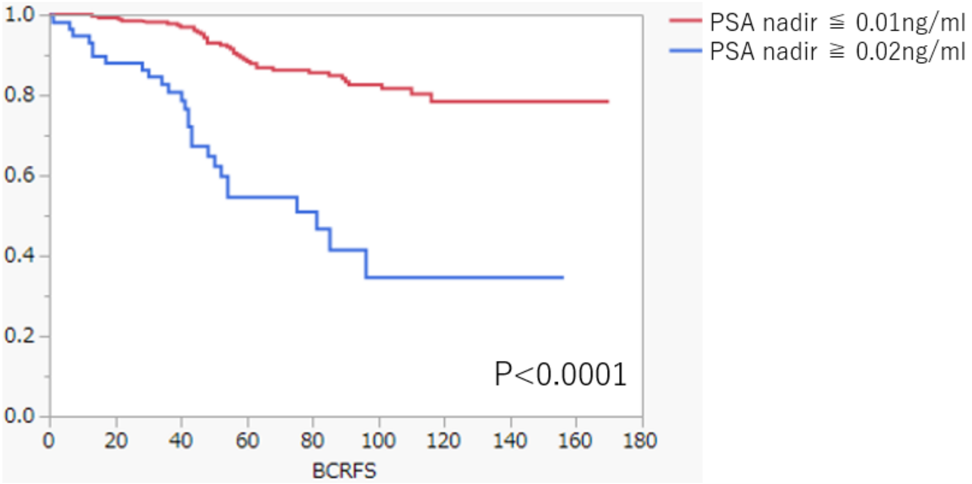

B) Progression free survival (PFS)

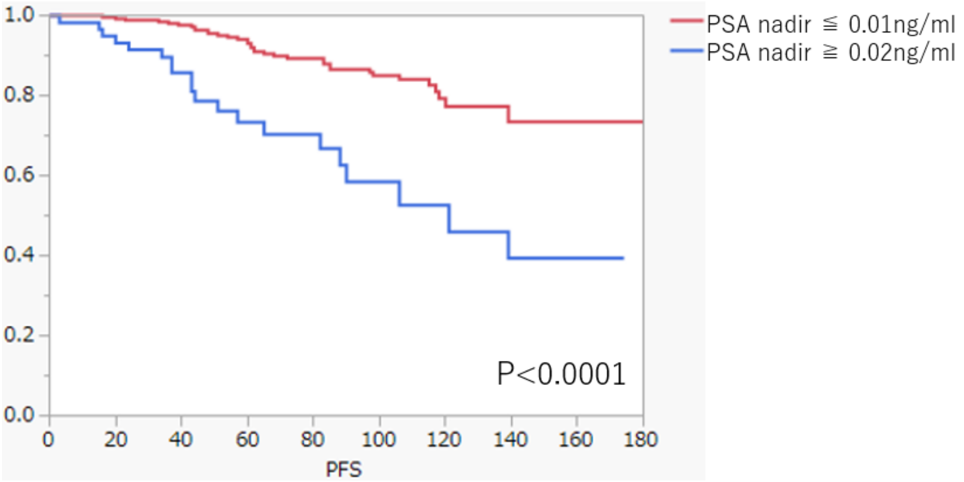

C) Overall survival (OS)

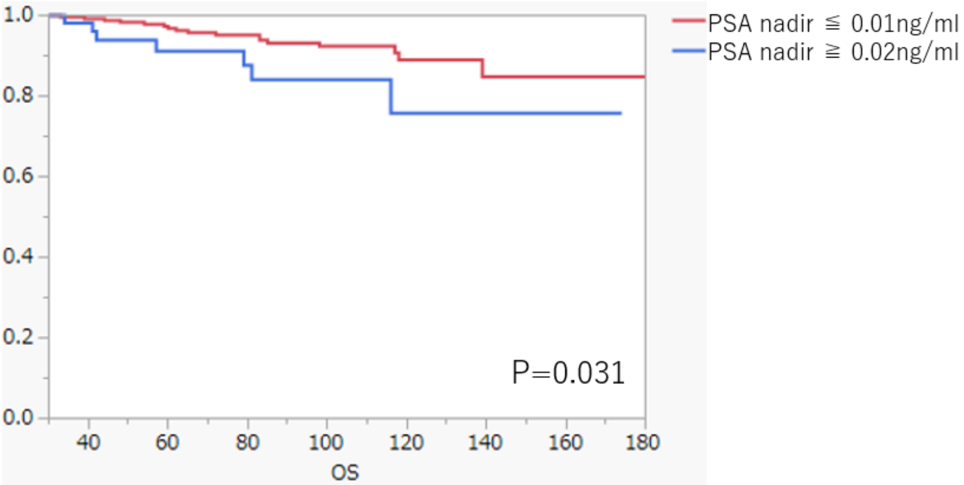

Supplement: Supplementary file 2 — Supplementary Figure 1 [file 66_2021_1784_MOESM2_ESM.pdf]
