# Supplementary material for: Trimodal therapy with high-dose-rate brachytherapy and hypofractionated external beam radiation combined with long-term androgen deprivation for unfavorable-risk prostate cancer
Source: Strahlenther Onkol. 2021 Apr 28;197(11):976–85. doi: 10.1007/s00066-021-01784-3 (PMC8547210; doi:10.1007/s00066-021-01784-3)

Supplementary Figure 2

Biochemical recurrence free survival (BCRFS) analysis based on time to PSA nadir (time to PSA nadir ≦6 months vs ≧7 months)

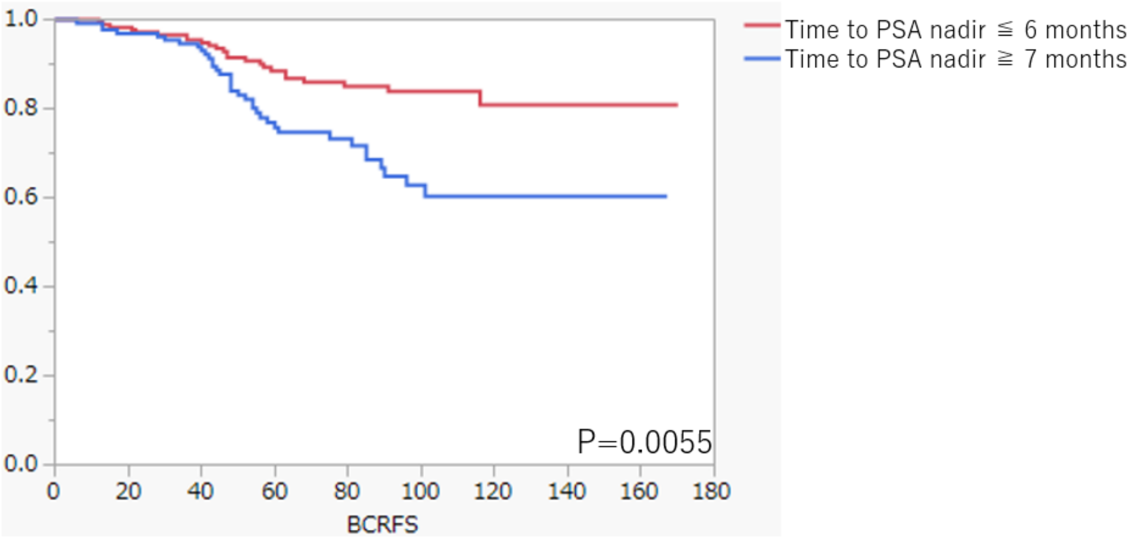

Supplement: Supplementary file 3 — Supplementary Figure 2 [file 66_2021_1784_MOESM3_ESM.pdf]
