# Supplementary material for: Trimodal therapy with high-dose-rate brachytherapy and hypofractionated external beam radiation combined with long-term androgen deprivation for unfavorable-risk prostate cancer
Source: Strahlenther Onkol. 2021 Apr 28;197(11):976–85. doi: 10.1007/s00066-021-01784-3 (PMC8547210; doi:10.1007/s00066-021-01784-3)

Supplementary Figure 3

Survival analysis based on protocol (very high risk [VHR] vs high risk [HR])

A) Biochemical recurrence free survival (BCRFS) (protocol 1)

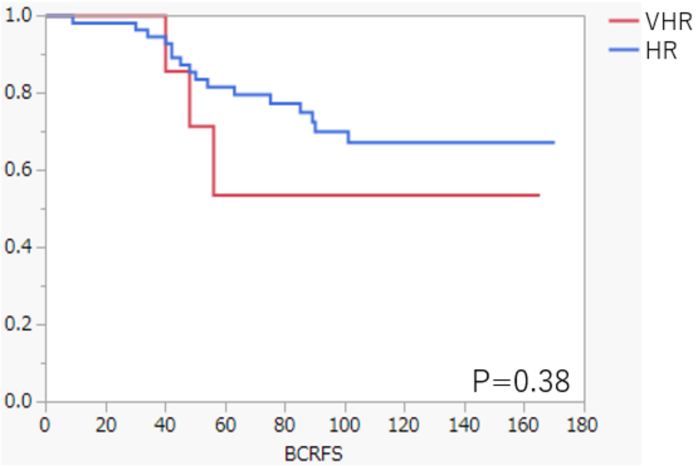

B) Progression free survival (PFS) (protocol 1)

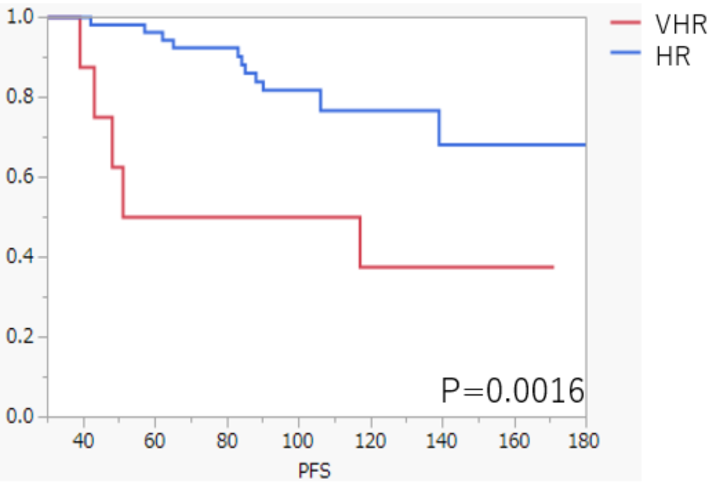

C) Overall survival (OS) (protocol 1)

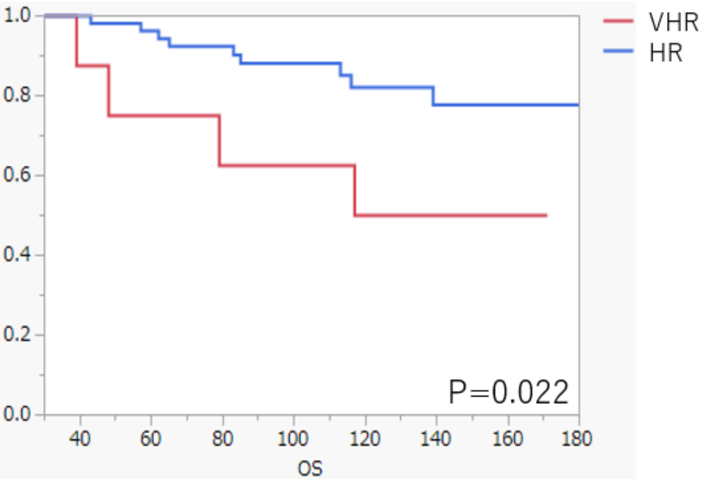

D) BCRFS (Protocol 2)

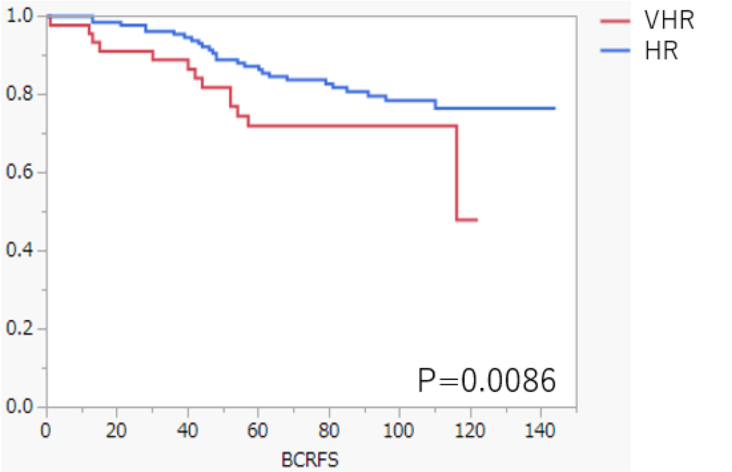

E) PFS (Protocol 2)

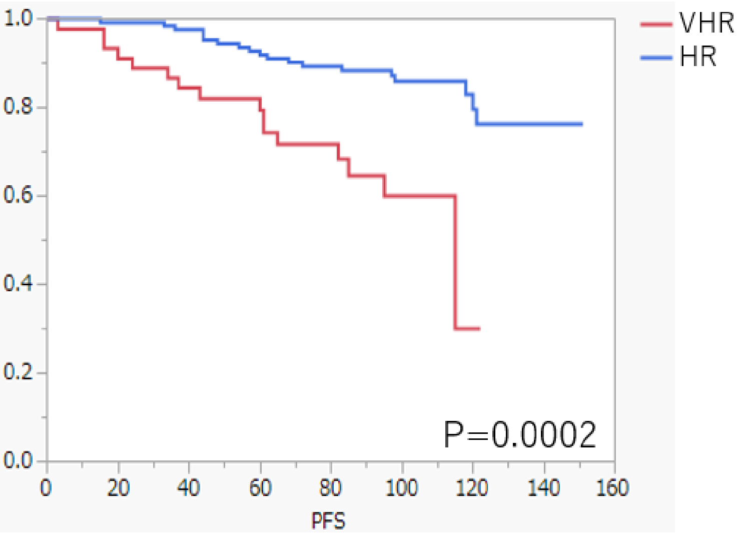

F) OS (Protocol 2)

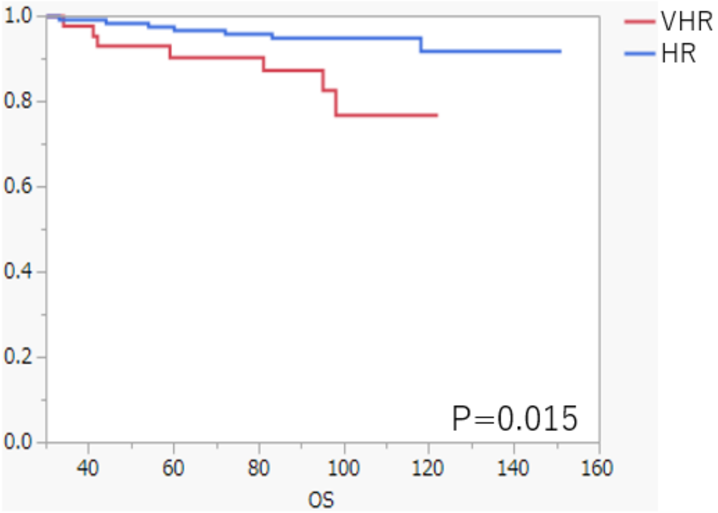

G) BCRFS (Protocol 3,4)

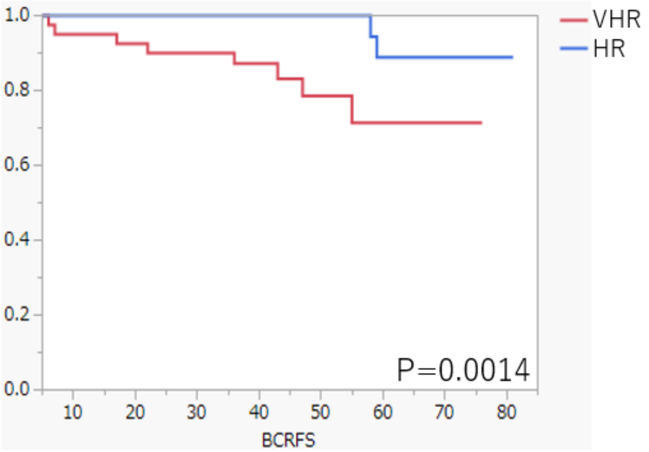

H) PFS (Protocol 3,4)

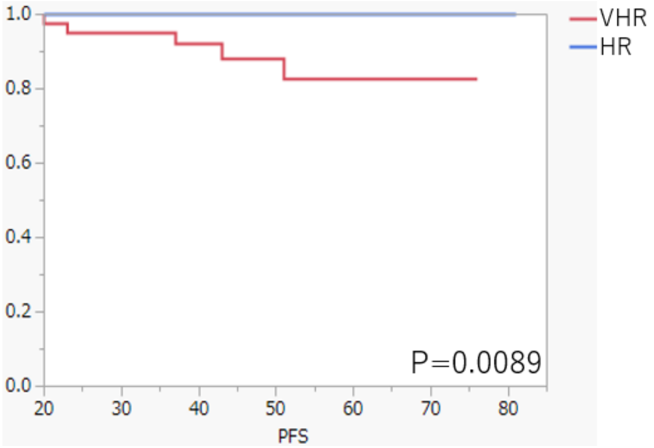

Supplement: Supplementary file 4 — Supplementary Figure 3 [file 66_2021_1784_MOESM4_ESM.pdf]
