# Supplementary material for: Trimodal therapy with high-dose-rate brachytherapy and hypofractionated external beam radiation combined with long-term androgen deprivation for unfavorable-risk prostate cancer
Source: Strahlenther Onkol. 2021 Apr 28;197(11):976–85. doi: 10.1007/s00066-021-01784-3 (PMC8547210; doi:10.1007/s00066-021-01784-3)
Supplement: Supplementary file 5 — Supplementary Table 2 [file 66_2021_1784_MOESM5_ESM.docx]

Supplementary Table 2

Patient demographics based on protocol

|  | ALL | Protocol 1 | Protocol 2 | Protocol 3 | Protocol 4 |
| --- | --- | --- | --- | --- | --- |
| N | 338 | 64 | 177 | 62 | 35 |
| Age | 69 (35-82) | 68 (59-82) | 71 (47-82) | 68 (35-80) | 67 (49-79) |
| Initial PSA  (ng/ml) | 25.09  (1.2-702.86) | 30.5  (4.1-201) | 25.27 (1.2-702.86) | 23.03 (4.51-211) | 15.16 (5.11-153.7) |
| Clinical T stage 3≧  (number) | 116 (34.3%) | 11 (17.2%) | 43 (24.3%) | 39 (62.9%) | 23 (65.7%) |
| Gleason score 8≧  (number) | 274 (81.1%) | 44 (68.8%) | 149 (84.2%) | 47 (75.8%) | 34 (97.1%) |
| PSA at HDR-BT  (ng/ml) | 0.08  (0.001-277.55) | 0.115  (0.001-277.55) | 0.07  (0.001-7.11) | 0.12  (0.001-211.63) | 0.105  (0.001-26.62) |
| PSA nadir  (ng/ml) | 0.001  (0.001-2.53) | 0.001  (0.001-2.53) | 0.001 (0.001-0.46) | 0.001 (0.001-0.39) | 0.01 (0.001-2.53) |
| Time to PSA nadir  (month) | 4 (0-85) | 9 (0-85) | 4 (0-75) | 5 (1-36) | 10 (0-27) |
| Very high risk  (number) | 93 (27.5%) | 8 (12.5%) | 45 (25.4%) | 25 (40.3%) | 15 (42.9%) |
| Follow up  (month) | 84 (30-180) | 116.5 (34-180) | 98 (31-151) | 55 (30-81) | 39 (30-47) |
| Abbreviations: HDR-BT; high-dose-rate brachytherapy, PSA; Prostate Specific Antigen  Values are expressed in median (range). | | | | | |
